# Working Memory Training Improves Emotion Regulation Ability

**DOI:** 10.1038/s41598-018-31495-2

**Published:** 2018-10-09

**Authors:** Lichao Xiu, Jie Wu, Lei Chang, Renlai Zhou

**Affiliations:** 1 Department of Psychology, Nanjing University, Nanjing, 210023 China; 2 School of Journalism and Communication, Beijing Normal University, Beijing, 100875 China; 3 Academy of psychology and Behavior, Tianjin Normal University, Tianjin, 300074 China; 4 Department of Psychology, University of Macau, Macau, S.A.R. China

## Abstract

Emotion regulation deficits are associated with various emotional disorders. Therefore, studies have attempted to improve emotion regulation ability to prevent psychopathological symptoms. Studies have revealed that working memory training—specifically attention control—can promote emotion regulation. In the present study, participants completed a running memory task in a 20-day training period. The participants’ pre- and post-test data on attention network functions and late positive potential (LPP) were assessed and analyzed. Compared with the control group, the training group’s orientation function improved markedly. In addition, LPP in relation to emotion regulation decreased during the 20-day training period. These results suggest that working memory training can improve emotion regulation ability, and the orientation function in the attention network may also contribute to this improvement.

## Introduction

Emotions play a key role in the daily lives of humans, and regulation of emotions affects quality of life. Therefore, researchers and practitioners have endeavored to improve humans’ emotion regulation ability to protect against emotional disorders^1^. Although developing effective intervention methods to promote emotion regulation is difficult^2^, promising studies have been conducted.

Wadlinger and Isaacowitz^3^ suggested that gaze pattern training is a valuable technique for promoting emotion regulation. This type of training, primarily conducted in the form of a dot-probe task, may modify the attention network functions of alerting and orientation. In addition, the dot-probe task requires one’s attention to be disengaged from negative information and reoriented toward positive or neutral information^3^. Gootjes, Franken, and Van Strien^4^ and Menezes *et al*.^5^ have investigated meditative practices as a means of promoting emotion regulation. Methods of emotion regulation involving meditative practices, including concentration meditation, mindfulness-based stress reduction, mindfulness-based cognitive therapy, and integrative body–mind training, likely require the entire attention network, which consists of the readiness and sustained attention component (alerting), selective attention (orientation), and inhibition of prominent distracters (executive control)^3,6–9^. Briefly, according to the Selection, Optimization, and Compensation with Emotion Regulation (SOC-ER) framework^10,11^, successful emotion regulation requires internal resources. In this framework, the frequently adopted internal resource refers to the ability to control one’s attention, and this ability is known as “working memory capacity”^10^.

Recent studies have indicated that working memory capacity, which is based on attention control, correlates with emotion regulation ability and relies heavily on the executive function processes in working memory^12^. Schmeichel, Volokhov, and Demaree^13^ reported that in a down-regulation task, participants with higher working memory capacity were more successful than were those with lower working memory capacity, and also experienced and expressed fewer emotional responses. In addition, the participants with lower working memory capacity were more susceptible to emotional contagion and less successful in applying suppression and reappraisal strategies. McRae, Jacobs, Ray, John, and Gross^14^ observed a positive correlation between individual differences in reappraisal ability and working memory capacity. A study based on emotional working memory training reported that individuals trained in emotion regulation exhibited markedly low levels of distress in response to negative images and considerable activity in the frontoparietal demand network^15^. These studies have established that working memory ability and emotion regulation are connected, and this connection is likely mediated by attention control.

Working memory is a universal processing ability, and its domain-general aspect is attention control^16,17^. Working memory capacity is used to process target-related information and eliminate not only the competitive response tendency but also interference from distractions^13^. According to Kane, Bleckley, Conway, and Engle^17^, working memory capacity refers specifically to attention control, whereas Fan and Posner^18^ proposed that it comprises the attention network functions, namely alerting, orientation, and executive control. The alerting function is responsible for the cognitive control of wakefulness and arousal, the orientation function is actively involved in information selection and filtering, and executive control is responsible for monitoring and conflict resolution. When competitive conflict occurs between task-relevant and task-irrelevant information, attention control (including the orientation and executive control functions) processes the information and provides a response favorable to the task-relevant information^19^.

Individuals with attention control deficit may have insufficient attention resources or an inadequate capacity for emotion regulation^20^. Cognitive control and attention are critical in the face of competition for limited resources^13^. Negative emotional stimuli are distinct, and thus are prioritized in resource processing. These stimuli, which are similar to distractions, may interfere with or block cognitive tasks^21,22^. For individuals with low attention control ability, attention resources primarily focus on negative emotional information, thereby not only exaggerating the effects of such information but also leading to failure of emotion regulation^23^. Individuals with lower attention control ability have greater difficulty handling isolated negative emotional distracters^24,25^. Moreover, studies on depression- and anxiety-related disorders have established that an exaggerated effect of negative information may facilitate the maintenance or exacerbation of depression and anxiety symptoms^26,27^.

Based on the aforementioned studies, we hypothesized that working memory capacity increased through training can enhance attention control ability, and thus may subsequently improve emotion regulation ability. In other words, attention control may be a shared component of and bridge between working memory and emotion regulation. Therefore, in the present study, a running memory task^28^ was performed and the attentional network test (ANT) was conducted to improve working memory and evaluate increases in attention control, respectively. In addition, we measured changes in emotion regulation and calculated correlations to assess the relationship between ANT components and emotion regulation outcomes.

In related studies, researchers commonly apply a specific emotional situation and ask participants to accomplish an emotion regulation task based on experimental manipulation. Subsequently, researchers analyze changes in participants’ emotion regulation ability based on changes in task indices (e.g., electrocardiogram, electroencephalography, and behavior performance results). In the present study, we selected a classic event-related potential (ERP) component, namely late positive potential (LPP), to assess its effect on emotion regulation. Generally, LPP amplitude is sensitive to emotional intensity^29,30^. Specifically, the higher the emotional intensity induced by a stimulus (emotional images were used as stimuli in the present study), the higher the LPP amplitude is. In addition, LPP amplitude decreases during emotion regulation^29^, and thus this phenomenon could be employed as an emotion regulation index^31,32^. Using these approaches, we conducted an ERP emotion regulation task where participants were required to view a set of emotional images and regulate their emotions. In the present study, it was expected that a decrease in LPP amplitude after a 20-day working memory training program would be observed and that the aforementioned three attention network functions would simultaneously undergo modification during training.

## Method

### Participants

Forty-two undergraduate and graduate students were recruited from local universities and were randomly divided into two groups. The average age of the training group was 22.45 ± 3.26 years (22 participants: 10 males and 11 females). The average age of the control group was 21.75 ± 3.00 years (20 participants: 5 males and 15 females). All participants were right-handed, had normal or corrected-to-normal vision, did not report any psychiatric disorders, and did not consume alcohol, tobacco, or any psychoanaleptics. The participants completed the Chinese Beck Anxiety Inventory (BAI) as well as the Beck Depression Inventory (BDI) before the experiment. Their scores are presented in Table 1. The participants in the two groups did not exhibit clinically significant symptoms of anxiety or depression. The participants signed an informed consent form before the experiment and were paid for their participation. The study procedures were approved by the sponsoring university’s institutional review board (Human Body Protection and Ethics Committee, School of Psychology, Beijing Normal University), and all the procedures for this study were performed in compliance with the principles of the Declaration of Helsinki.

**Table 1:** Age, BAI (Beck Anxiety Inventory) and BDI (Beck Anxiety Inventory) of the training and control group.

	Training Group (*N* = 22)	Control Group (*N* = 20)
male	10	5
female	12	15
age	22.45 ± 3.26	21.75 ± 3.00
BAI	27.86 ± 5.75	27.00 ± 4.70
BDI	8.09 ± 6.44	4.65 ± 4.20

### Stimuli and Procedure

A total of 132 images (99 negative, 33 neutral) were chosen from the Chinese Native International Affective Picture System^33^. Five neutral images and 15 negative images were used as the practice block. The 112 remaining images were divided into four blocks of 28 images (7 neutral images and 21 negative images per block). For neutral images, the valence was 5.17 ± 1.57, and the arousal was 4.70 ± 1.77. For negative images, the valence was 2.82 ± 2.14, and the arousal was 5.07 ± 2.17. In particular, for the arousal score, there was no difference between all the four conditions: *F* (3, 131) = 2.323, *p* = 0.078.

The training and control groups both completed the ANT and emotion regulation tasks one day before training (pretest) and the day after training (posttest). In addition, the training group dedicated 20–30 minutes daily to complete the 20-day updating function training program called the running memory task^28^. The control group did not participate in the training. The time interval between the pretest and posttest was 20 days.

#### Pretest and posttest

The ANT-short version (ANT-S) was used to measure attention control ability^34,35^. An image depicting a central target (an arrow pointing left or right) flanked on each side by two arrows was presented. The central arrow and the flanked arrows pointing in the same direction was the congruent condition. The opposite scenario (i.e., arrows pointing in different directions) was the incongruent condition. Three cue conditions were provided: (1) no cue (only a central fixation “+” was presented), (2) central cue (a central asterisk cue “*” was presented), and (3) spatial cue (the cue was presented in the location where the central target arrow would be presented, either above or below the central fixation). In the task, the participants compared whether the central arrow pointed left or right by clicking on the left or right buttons of a mouse. The experiment comprised 144 trials, divided into 3 blocks. Results were separately tabulated on the basis of the reaction time under various experimental conditions to obtain scores for the three attention network functions: alerting (no cue minus central cue condition), orienting (central cue minus spatial cue condition), and executive control (congruent minus incongruent condition). The ANT-S scores were analyzed using ANOVA on SPSS 18.0.

The participants completed an emotion regulation task, and their EEG data were simultaneously recorded. This task was similar to the tasks adopted in other classic studies^36^. The participants watched the emotional images and performed the appropriate task: Watch (neutral images), Attend (negative images), Distract (negative images), or Reappraise (negative images). The Watch and Attend conditions were functionally equivalent because both required the participants to simply pay attention to the images. In the Distract condition (specifically, visual attentional deployment), the participants focused on the emotion-unrelated content in the images, such as picture composition, lines, and scenes. In the Reappraise condition, the participants adopted a neutral attitude toward understand the content of the image or to reconstruct the image meaning, for example, imagining the emotional scenes in the image are actors or stage properties. Each block contained 7 trials of each condition, and the sequence of trials was randomized for each participant. Moreover, the sequence of the blocks was counterbalanced for different participants. In addition, arrangement of the negative image for the Watch, Distract, and Reappraise conditions varied across participants, ensuring that each negative image appeared once in each condition.

**Figure 1:**
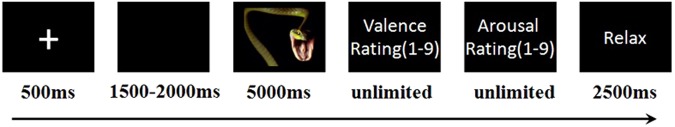
Trial structure of the emotion regulation task (an example of a WATCH trial). Note: The example snake image was obtained from Liu, Xu, and Zhou^33^.

The trial structure is presented in Fig. 1. First, a colorized “+” cue was presented for 500 ms. The color of the cues prompted the participants to perform the required tasks after seeing the image: white for Watch, gray for Attend, blue for Distract, and green for Reappraise. After the cue, a blank screen was displayed for 1.5–2 s, followed by an image for 5 s. When the image disappeared, the participants were asked to rate their level of valence and arousal on a scale of 1–9 (for valence rating, the “1” represents the most negative, and the “9” represents the most positive; for arousal rating, the “1” represents the most calming and the “9” represents the most exciting). The next trial began after a 2.5-s interval. To perform the emotion regulation task effectively, the participants practiced until they remembered the instructions for each cue.

#### Training task

The training program comprised a running working memory task, which was used by Zhao *et al*.^28^, with three versions using different materials: letters, animals, and locations. For example, in the letter running working memory task, a fixation “+” was initially presented in the center of the screen to indicate the onset of the task. Thereafter, several letters were successively presented. The number of presented letters was randomly determined and varied across trials, with 5, 7, 9, or 11 letters presented in each trial. The participants sequentially remembered the final three letters presented. Finally, the screen went blank, and the participants entered the final three letters presented in the sequence using a keyboard. Each letter was presented for 1750 ms, and the difficulty level of the task was subsequently changed according to the scores of the participants: as the scores increased, the presentation duration was decreased. The participants completed 6 blocks with 5 trials in this task. If the participants responded correctly to 3 or more trials, the presentation duration for each letter in the next block was decreased by 100 ms. By contrast, if the participants responded incorrectly to 2 or more trials, the presentation duration of each letter was increased by 100 ms. A given day’s training was based on the previous day’s record. The other two versions of the task were similar to the afore described version (refer to Zhao *et al*.^28^ for additional details).

During training, the participants completed all three computerized training programs once a day in the laboratory, with no time constraints and in their preferred sequence. The time interval between the pretest and posttest for both groups was 20 days.

### EEG Recording and Quantification

EEG data were recorded using a Neuroscan NuAmps 40 channel DC amplifier at 1000 Hz. The EEG data was band-pass filtered from 0 to 100 Hz. The electrode impedances were maintained at less than 5 kΩ, and the EEG was recorded using 40 Ag/AgCl electrodes mounted on a custom-made cap that used an extended 10–20 system. The AFz electrode served as the ground, and the left mastoid was referenced online. However, the EEG signal was re-referenced to the average of the bilateral mastoid electrodes offline.

Offline analysis was performed using the Scan 4.5 and EMSE 5.5.2 software, and blink artifacts were eliminated using ICA method. EEG signals were low-pass filtered at 30 Hz. EEG epochs were segmented for a duration that began 200 ms before image onset and continued for 5000 ms and was baseline corrected with a 200 ms pre-stimulus period. Epochs more than 150 *μV* as artifacts were excluded to ensure that each experimental condition had more than 30 effective trials. According to previous studies^30,36,37^, the LPP amplitude is maximum at the Pz site. Therefore, the LPP mean amplitude data (500–2000 ms after image onset) of the Pz electrode was extracted for statistical analysis using SPSS 18.0. The Greenhouse-Geisser correction was applied to the *p* values.

Finally, the correlation coefficients for the increments (posttest – pretest) of the three components of ANT, the subjective emotion rating, and the LPPs in the Distract and Reappraise conditions were calculated. That is due to the limitations of experimental design and sample size. There is no suitable mediating effect model to be used in this study, and therefore, correlation analysis is used as an alternative.

## Results

### Working Memory Capability

**Figure 2:**
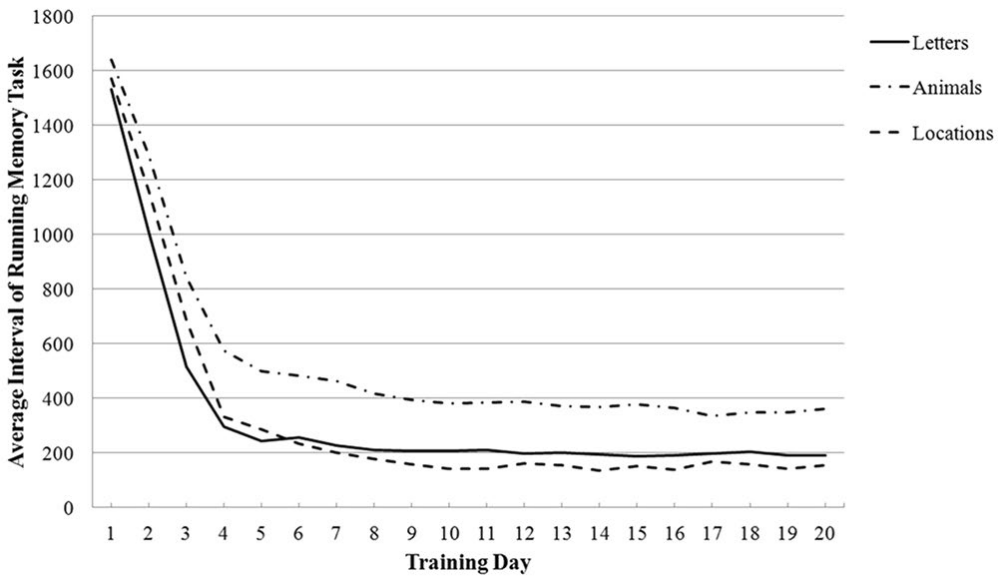
The three running memory task performance trends of training group over time.

The gradual trends in the three running memory task performances of the training group are presented in Fig. 2. The vertical axis indicated the time interval between the letters (or animals or locations) of the updating memory task. When the interval was shorter, the task was more difficult. The difficulty level of the task increased as the training progressed. The training task was adaptive. Participants who responded correctly at the first stage experienced increased difficulty levels in the subsequent stages.

In order to compare the effects of training, we used the performance of Day 1 and Day 20 to measure working memory ability and conducted the paired t-test on the three versions. All the effects were significant: on letters: *t* (21) = 88.484, *p* < 0.001, Cohen’s *d* = 21.790; on animals: *t* (21) = 34. 842, *p* < 0.001, Cohen’s *d* = 9.52; on locations: *t* (21) = 69.755, *p* < 0.001, Cohen’s *d* = 15.127. Therefore, these results revealed that the training performance of the participants increased.

### Attention Network Test

The performances of both groups in the ANT task were presented in Table 2. The three components of attention network function were separately analyzed using mixed-model ANOVA with time (pretest vs. posttest) as the within-participant factor and training (control vs. training) as the between-participants factor.

**Table 2:** Performance of Pre and Post-Tested Attentional Network Test Task (ms) (M ± SD).

Experimental Conditions	Training Group (*N* = 22)		Control Group (*N* = 20)	
	Pre-Training	Post-Training	Pre-Training	Post-Training
No cue	547.70 ± 67.91	531.85 ± 67.78	529.92 ± 49.73	526.87 ± 54.76
Central cue	519.81 ± 65.75	502.17 ± 63.19	508.46 ± 51.44	497.86 ± 49.54
Spatial cue	483.55 ± 63.45	451.34 ± 60.49	467.49 ± 49.35	462.17 ± 60.19
Congruent	480.61 ± 59.94	464.94 ± 58.00	466.15 ± 43.33	464.58 ± 46.66
Incongruent	553.18 ± 68.47	525.13 ± 68.55	538.61 ± 59.33	526.58 ± 59.19
Alerting	27.89 ± 27.48	29.68 ± 22.37	21.46 ± 21.76	29.01 ± 23.15
Orienting	36.26 ± 22.10	50.83 ± 22.34	40.97 ± 23.11	35.69 ± 24.11
Executive control	72.58 ± 19.06	60.19 ± 21.95	72.47 ± 31.08	62.00 ± 15.94

For the alerting function, the main effect of time was nonsignificant, *F* (1, 40) = 0.497, *p* = 0.485, *η*^2^_*P*_ = 0.012. The main effect of training was nonsignificant, *F* (1, 40) = 0.075, *p* = 0.786, *η*^2^_*P*_ = 0.002, with nonsignificant interaction of time (pretest, posttest) × training (control, training), *F* (1, 40) = 0.062, *p* = 0.805, *η*^2^_*P*_ = 0.002.

For the orienting function, the main effect of time was nonsignificant, *F* (1, 40) = 1.154, *p* = 0.289, *η*^2^_*P*_* = *0.028, and the main effect of training was nonsignificant, *F* (1, 40) = 0.869, *p* = 0.357, *η*^2^_*P*_ = 0.021. However, we observed a significant interaction of time (pretest, posttest) × training (control, training), *F* (1, 40) = 5.269, *p* = 0.027, *η*^2^_*P*_* = *0.116. The simple effect analysis indicated nonsignificant difference between the two groups at the pretest (*F* (1, 40) = 0.456, *p* = 0.504), however, a significant difference was observed posttest (*F* (1, 40) = 4.465, *p* = 0.041). That is to say, although the orienting performance and function of the training group were significantly higher than those of the control group after training, the orienting function of the two groups were not significantly different before training.

For the executive control function, the main effect of time was significant, *F* (1, 40) = 7.896, *p* = 0.008, *η*^2^_*P*_ = 0.165. However, the main effect of training was nonsignificant, *F* (1, 40) = 0.022, *p* = 0.882, *η*^2^_*P*_ = 0.001. In addition, we observed nonsignificant interaction of time (pretest, posttest) × training (control, training), *F* (1, 40) = 0.056, *p* = 0.815, *η*^2^_*P*_ = 0.001.

Therefore, only orienting function have shown the training effect.

### Subjective Emotion Ratings

Because we were primarily concerned with the changes in emotion regulation ability, we compared only the three negative image conditions in the emotion regulation task: Attend, Distract, and Reappraise. Moreover, we used a mixed-model ANOVA with time (pretest vs. posttest) and condition (Attend vs. Distract vs. Reappraise) as the within-participant factor and training (control vs. training) as the between-participants factor (Table 3).

**Table 3:** Rating Scores and Late Positive Potential Amplitudes (μV) of Pre- and Post-Tested Emotion Regulation Task (M ± SD).

	Training Group (*N* = 22)		Control Group (*N* = 20)	
	Pre-Test	Post-Test	Pre-Test	Post-Test
**Arousal**				
Attend	4.53 ± 1.35	4.82 ± 0.96	5.11 ± 1.38	5.00 ± 1.51
Distraction	4.22 ± 1.26	4.57 ± 1.07	5.07 ± 1.49	4.83 ± 1.41
Reappraise	4.49 ± 1.50	4.62 ± 1.14	5.26 ± 1.23	4.95 ± 1.44
**Valence**				
Attend	3.25 ± 0.85	3.42 ± 0.87	2.98 ± 0.88	3.27 ± 1.07
Distraction	3.55 ± 0.94	3.72 ± 0.94	3.06 ± 1.01	3.33 ± 1.02
Reappraise	3.43 ± 0.97	3.51 ± 0.77	3.04 ± 0.98	3.33 ± 1.12
**Late Positive Potential**				
Attend	5.64 ± 8.56	7.62 ± 7.05	4.24 ± 8.08	4.60 ± 8.19
Distraction	2.83 ± 5.60	1.61 ± 7.61	3.61 ± 6.56	6.01 ± 5.26
Reappraise	6.41 ± 5.06	3.31 ± 5.37	3.53 ± 7.56	7.75 ± 6.53

Note: The evaluation of valence and arousal were given by participants in an emotion regulation task on a 9-point scale with 1 representing the most negative and 9 representing the most positive for the valence score, and with 1 representing the most calming and 9 representing the most exciting for the arousal score.

For the arousal score, the main effect of time was nonsignificant, *F* (1, 40) = 0.018, *p* = 0.893, *η*^2^_*P*_ < 0.001. The main effect of training was nonsignificant, *F* (1, 40) = 1.802, *p* = 0.187, *η*^2^_*P*_ = 0.043. The main effect of condition was significant, *F* (2, 80) = 5.463, *p* = 0.007, *η*^2^_*P*_ = 0.120. In addition, we compared the three levels of conditions using the Bonferroni method and observed significant differences between Attend and Distract (*p* = 0.015) as well as between Distract and Reappraise (*p* = 0.018). However, nonsignificant differences were observed between Attend and Reappraise (*p* = 1.000). The interaction of time × condition was nonsignificant, *F* (2, 80) = 1.244, *p* = 0.294, *η*^2^_*P*_ = 0.030. The interaction of time × training was nonsignificant, *F* (1, 40) = 2.598, *p* = 0.115, *η*^2^_*P*_ = 0.061. The interaction of condition × training was nonsignificant, *F* (2, 80) = 1.307, *p = *0.276, *η*^2^_*P*_ = 0.032. Moreover, the interaction of time × condition × training was nonsignificant, *F* (2, 80) = 0.324, *p* = 0.685, *η*^2^_*P*_ = 0.008. In other words, the arousal score under Distract condition is significantly lower than the condition of Attend and Reappraise, but the difference in arousal score between Attend and Reappraise conditions is not significant, whether or not it has been trained.

For the valence score, the main effect of time was significant, *F* (1, 40) = 5.234, *p* = 0.028, *η*^2^_*P*_ = 0.116. The main effect of condition was significant, *F* (2, 80) = 3.521, *p* = 0.037, *η*^2^_*P*_ = 0.081. In addition, we compared the three levels of conditions using the Bonferroni method and observed nonsignificant differences between Attend and Distract (*p* = 0.066). Nonsignificant differences were observed between Distract and Reappraise (*p* = 0.453) as well as between Attend and Reappraise (*p* = 0.537). The main effect of training was nonsignificant, *F* (1, 40) = 1.383, *p* = 0.247, *η*^2^_*P*_ = 0.033. The interaction of time × condition was nonsignificant, *F* (2, 80) = 0.174, *p* = 0.840, *η*^2^_*P*_ = 0.004. The interaction of time × training was nonsignificant, *F* (1, 40) = 0.607, *p* = 0.441, *η*^2^_*P*_ = 0.015. The interaction of condition × training was nonsignificant, *F* (2, 80) = 1.385, *p* = 0.256, *η*^2^_*P*_ = 0.033. Moreover, the interaction of time × condition × training was nonsignificant, *F* (2, 80) = 0.288, *p* = 0.749, *η*^2^_*P*_ = 0.007. That is, the valence score of the posttest is significantly lower than that of the pretest, regardless of training or use of the emotion regulation strategy. There were significant differences between the three conditions (Attend, Distract, and Reappraise) in the overall effect on the valence score, but there was no difference between the two.

In combination with the above results, the distraction strategy significantly reduced the arousal of negative emotions, and the reappraise condition showed nonsignificant training effect on behavior.

### LPP

**Figure 3:**
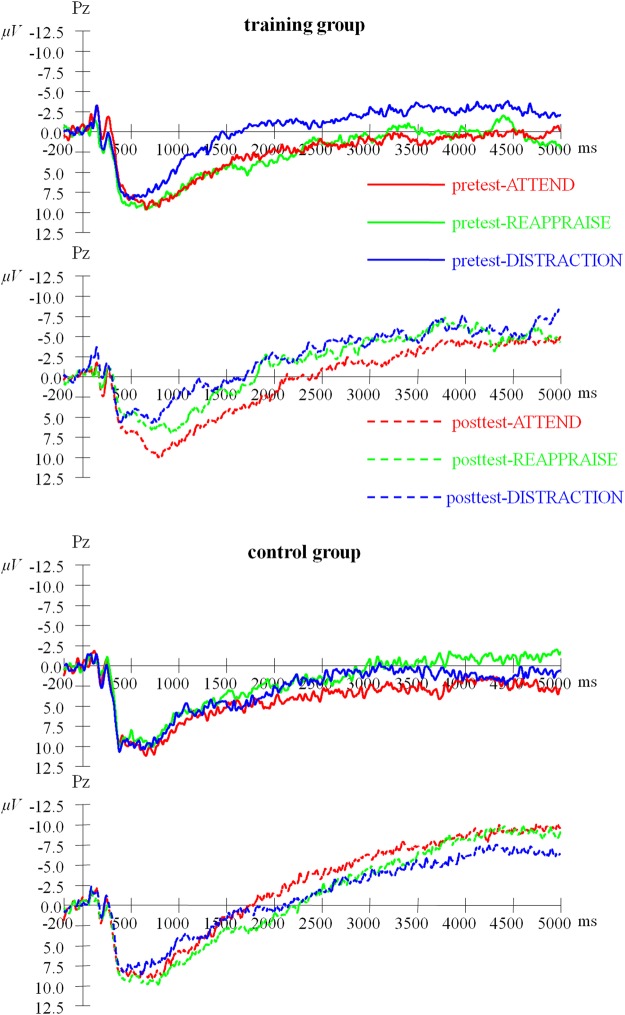
Grand average LPPs at Pz in the emotion regulation task for the training group (upper graph) and control group (bottom graph).

The average LPPs are presented in Fig. 3. A mixed-model ANOVA with time (pretest vs. posttest) and condition (Attend vs. Distract vs. Reappraise) as the within-participant factor and training (control vs. training) as the between-participants factor yielded a significant main effect of condition (Table 3), *F* (2, 80) = 3.517, *p* = 0.042, *η*^2^_*P*_ = 0.081. However, there was a nonsignificant main effect of time, *F* (1, 40) = 0.264, *p* = 0.610, *η*^2^_*P*_ = 0.007, and of training, *F* (1, 40) = 0.058, *p* = 0.811, *η*^2^_*P*_ = 0.001. In addition, the interaction of time × condition, (*F* (2, 80) = 0.170, *p* = 0.839, *η*^2^_*P*_ = 0.004), and time × training, (*F* (1, 40) = 1.067, *p* = 0.308, *η*^2^_*P*_ = 0.026), were nonsignificant. However, the interaction of condition × training was significant, *F* (2, 80) = 4.336, *p* = 0.022, *η*^2^_*P*_ = 0.098. Moreover, the interaction of time × condition × training was significant, *F* (2, 80) = 7.356, *p* = 0.001, *η*^2^_*P*_ = 0.155. Therefore, we performed the simple main effect tests and established that the two groups had nonsignificant differences in the Attend, Distract, and Reappraise conditions at the pretest: *F* (1, 40) = 0.30, *p* = 0.589; *F* (1, 40) = 0.17, *p* = 0.684; and *F* (1, 40) = 0.59, *p* = 0.447, respectively. In the posttest, the two groups had significant differences in the Distract condition, *F* (1, 40) = 4.65, *p* = 0.037, and in the Reappraise condition, *F* (1, 40) = 5.84, *p* = 0.020. However, nonsignificant difference was observed in the Attend condition, *F* (1, 40) = 1.65, *p* = 0.206. In other words, at the pretest, the LPP amplitude of the two groups showed no significant difference under the conditions of Attend, Distract and Reappraise. However, at the posttest, the LPP of the two groups did not differ significantly under Attend condition, and there was significant difference in both Distract and Reappraise conditions: the LPP amplitude of the training group was less than that of the control group.

For the sake of clarity, we calculated their increments by the posttest minus the pretest as the dependent variable for statistics (Table 4). There was a nonsignificant main effect of condition, *F* (2, 80) = 0.170, *p* = 0.844, *η*^2^_*P*_ = 0.004; in addition, there was a nonsignificant main effect of group, *F* (1, 40) = 1.067, *p* = 0.308, *η*^2^_*P*_ = 0.026. However, the interaction of condition × group was significant, *F* (2, 80) = 7.356, *p* = 0.001, *η*^2^_*P*_ = 0.155. The simple effects analysis suggested that on training group, the LPP amplitude changes (mainly reduction) in Reappraise condition was larger than Attend (*p* = 0.005), but no difference with Distract (*p* = 0.859), and no difference between Distract and Attend (*p* = 0.157). Moreover, on control group, there were nonsignificant among these three conditions (Attend vs. Distract: *p* = 0.691; Attend vs. Reappraise: *p* = 0.058; Reappraise vs. Distract: *p* = 0.964). These results suggest that compared with the control group, the LPPs of the training group were reduced by updating ability training (running memory training task), which indicated an improvement in emotion regulation ability of training group.

**Table 4:** Increments of LPP Amplitudes of Emotion Regulation Task (M ± SD).

	Training Group (*N* = 22)	Control Group (*N* = 20)
Attend	1.98 ± 5.90	0.36 ± 5.96
Distraction	−1.23 ± 6.68	2.40 ± 6.24
Reappraise	−3.09 ± 6.87	4.22 ± 6.94

Finally, significant correlations were established between orienting increment and subjective valence scores in the Attend (*r* = 0.456, *p* = 0.002), Distract (*r* = 0.372, *p* = 0.015), and Reappraise (*r* = 0.449, *p* = 0.003) conditions. However, the association between orienting increment and subjective arousal score was not statistically significant in the Attend (*r* = −0.139; *p* = 0.378), Distract (*r* = −0.010; *p* = 0.952) or Reappraise (*r = *−0.060; *p* = 0.704) conditions. All the others correlation coefficients were not significant.

All correlations between subjective emotion rating and the decrease of LPPs were not significant (for valence: Distract: *r* = −0.032, *p* = 0.841; Reappraise: *r* = −0.045, *p = *0.776; for arousal: Distract: *r* = 0.213, *p* = 0.175; Reappraise: *r* = 0.103, *p* = 0.514). All the correlation coefficients were shown in Table 5.

**Table 5:** Correlations Between Attentional Network Test Components and Emotion Regulation Measures.

	Alerting Increment	Orienting Increment	Executive Control Increment
Valence Score Increment in Distraction	0.237	0.372*	0.093
Valence Score Increment in Reappraisal	0.156	0.449*	−0.010
Arousal Score Increment in Distraction	0.053	−0.010	0.041
Arousal Score Increment in Reappraisal	0.212	−0.060	−0.036
LPP Decrease in Distraction	−0.128	−0.066	0.067
LPP Decrease in Reappraisal	0.033	−0.067	0.071

Note: The increments of all the emotion regulation scores (valence, arousal, and LPP amplitude) under two conditions (distraction and re-appraisal) were calculated by subtracting pre-test from post-test. *p < 0.05.

## Discussion

We attempted to improve emotion regulation ability through working memory training. Several valuable results were obtained. First, we established that the individual orientation function of the attention network had improved after 20 days of working memory training. Second, as the main indicator of emotion regulation, the posttraining LPP amplitude exhibited an apparent decline, which was indicative of an improvement in individual emotion regulation ability. Third, a significant correlation was observed between the orientation function and the subjective aspect of emotion regulation. These findings may indicate a connection between emotion regulation and attention control.

The primary finding of this study was that training positively influenced the orientation function and may simultaneously enhance emotion regulation ability. Based on our results, we propose that the mechanism likely operates as follows. The orientation function is the attention network mechanism that selects and filters external information, including the focus of attention engagement, attention shift, and attention disengagement processes^38^. In the emotion regulation choice framework^39^, emotion regulation comprises two stages, namely the attentional selection stage and semantic meaning stage, both of which are resource limited and require attention control. In the initial attentional selection stage, information can be disengaged from negative processing and undergo neutral and emotionally irrelevant processing, which is typically called “distraction.” In the subsequent semantic meaning stage, reappraisal is required to modify the primary emotional meaning through semantic processing and update it to form a new semantic meaning. However, working memory capacity affects the selection and allocation of resources, which the orientation function is responsible for. In other words, within the framework of emotion regulation, working memory training may involve an ever-present component, namely orientation, which performs information selection and filtering. By contrast, the alerting and executive control functions may not be involved. Bleckley, Foster, and Engle^40^ demonstrated that individuals with a relatively high working memory capacity focus on objects in need of attention, whereas individuals with a relatively low working memory capacity apply focus in a more dispersive manner.

By training working memory capacity (updating ability in the present study), the orientation function was enhanced. Through this method, allocation of an individual’s attentional resources can be optimized. In other words, when faced with negative events, individuals can disengage their attention from negative information, provide timely updates of emotional information in working memory, break the repetitive process of maladaptive thinking, and attenuate rumination involving negative emotions^41,42^. Thereafter, attention can be focused on processing events in daily life to improve emotional experiences. Studies have demonstrated that weak orientation function can predict an increase in rumination tendency, which is a depressive symptom^43^. In addition, a significantly negative correlation exists between the orientation function and negative emotions^38^. Regarding alerting and executive control, these functions are concerned with achieving and maintaining an alert state and resolving conflict among responses^18^. However, these functions may not be as sensitive to working memory training as is orientation, and thus failed to exhibit training outcomes in the present study.

Researchers have attempted to improve emotion regulation ability and affective states through attentional bias training. For example, attentional training has been used to treat symptoms of anxiety and depression in clinics^44,45^. Watson and Purdon^46^ defined attentional training as involving selective attention, attention switching, and divided attention. This type of training primarily concerns the selection and allocation of attention, which is dependent on the orientation function. Moreover, attentional training is fundamentally similar to the dot-probe task performed in the present study. Some studies have conducted meditation training to improve attention function, emotion regulation ability, and affective experience^47^. Meditation training can improve allocation and regulation abilities by shifting the focus of attention^6,8,9^. As mentioned, attention control may be the basis of these training mechanisms. Similar to the cited studies, the present study reported that the description of working memory training as facilitating improvements in emotion regulation ability is based on the attention function. This implies that emotion regulation ability improvement should focus on the attention function, and diverse training methods may have common mechanisms. The selection and allocation of attention may be a common method of various training mechanisms, and different types of training may be suitable for different individuals. These results support the SOC-ER framework^10^. Moreover, because little evidence on the use of attention training in clinical settings is available, we suggest that future studies strive to confirm our aforementioned hypothesis and attempt to determine which form of attention training is the most effective. In our opinion, any form of training that can improve attention deployment or at least improve the orientation function is promising.

Our study established that working memory training should focus on distraction and reappraisal strategies. These two emotion regulation strategies have positive effects that are primarily reflected by the decrease in LPP amplitude. However, in practice, working memory training seems to have a slightly stronger influence on the reappraisal strategy than on the distraction strategy (a training group increment comparison revealed that the reappraisal strategy reduced the LPP to a greater extent than did the distraction strategy). LPP amplitude represents the intensity of an emotional reaction and can be used to measure the effectiveness of emotion regulation changes as an LPP index^32^. The aforementioned finding regarding the influence of working memory training may have been a result of the process adopted in reappraisal being more complex and high ordered than that adopted by distraction. Reappraisal involves an individual rethinking the meaning of emotional stimuli or events and changing their effects^48^. Moreover, reappraisal requires a transfer of emotional representation^49^, use of language and memory^50^, and management and monitoring of other processes^51^. In addition, the number of brain regions involved in reappraisal is greater than that involved in distraction, and most regions functionally overlap with working memory^52^. By contrast, distraction—specifically visual attention deployment, which is related to and represents attention and emotion regulation strategies—requires only focus and attention switching^53^. Before undergoing working memory training, an individual faces difficulty using the reappraisal strategy, whereas using the distraction strategy is easy. After training, an individual’s ability to use the reappraisal strategy is increased and it becomes comparable to his or her increased ability to use the distraction strategy.

The present study verified that the orientation function was significantly correlated with the subjective valence aspect of emotion regulation but not with the decrease in LPP. Moreover, all correlations between subjective emotion ratings and decreased LPP were nonsignificant. These findings may have resulted from the training not being sufficiently rigorous to completely change the participants’ emotional experiences. However, the program was rigorous enough to change their physiological indices. Generally, subjective experiences and physiological indices are both measures of emotion, but they represent different aspects of emotion regulation. However, a change in physiological index does not always imply that one’s subjective experience has been altered, and neither is the opposite true.

Our results partially supported our hypothesis because the interrelationships between working memory updating, the attention orientation function, and emotion regulation (subjective experience and physiological indices) are highly complex. We determined that working memory updating training can improve the ability to regulate emotions and enhance the orientation function of attention control. However, no robust evidence to determine the relationship between orientation and emotion regulation (LPP) was obtained. We also observed relationships of orientation with some aspects of emotion regulation (subjective valence rating). In summary, there remains considerable scope for further exploration.

In conclusion, the present study focused on whether working memory training can improve emotion regulation ability. The primary difference between the two groups in this study was whether training was provided within a 20-day period. Studies have advocated establishing an “active” control group in working memory training research^54^. By contrast, the present study explored whether working memory training can promote emotion regulation. A blank control group was established primary in light of the problems associated with active control groups. In such a 20-day training period (which is relatively long), emotions can easily be affected by various factors. If we had conducted an active task, the participants would have experienced boredom, which would have negatively affected their baseline conditions for emotion regulation. Furthermore, comparing the effects of training would have been difficult, and the potential cost of the experiment would have been extremely high. Therefore, we selected a relatively conservative and reliable design, established a blank control group, and did not create an activity task control group. We obtained noteworthy results that supported our conclusion to a certain extent. Moreover, we recruited healthy participants. To help solve psychiatry problems, future research must involve participants with psychiatric disorders because some such disorders are associated with emotion regulation deficits. For instance, posttraumatic stress disorder symptoms are significantly associated with emotion regulation difficulties^55^, and spontaneous emotion regulation difficulties are a crucial factor of vulnerability to depression^56^. In addition, impaired emotion regulation can predict anxiety disorders^57^. Nevertheless, the results of this study verified that working memory training not only had a positive effect on emotion regulation ability but also improved the orientation function of the attention network. These outcomes suggest that working memory training could extend to other abilities related to attention control and emotion regulation. Future studies should explore training methods that can effectively promote the orientation function. Such promotion could lead to more effective interventions for emotion regulation. The findings of the present study deepen our understanding of the emotion regulation process. Moreover, this research facilitates comprehension of the interrelationships between working memory training, attention regulation, and emotion regulation.
